# The Medical Society of the County of Erie

**Published:** 1909-08

**Authors:** Franklin C. Gram


### SOCIETY PROCEEDINGS.

### The Medical Society of the County of Erie.

Reported by FRANKLIN C. GRAM, M. D., Secretary.

*Monday, June 21, 1909.*

THE regular meeting of the Medical Society of the County of Erie was held at the University Club on Monday evening, June 21, 1909.

The President, Dr. Charles A. Wall, called the assembly to order at 9 o'clock.

The minutes of the last regular meeting held April 19, 1909, and the minutes of the council meetings, held May 3, 1909, June 4, 1909, and June 21, 1909, were approved. Treasurer Lytle read the list of those who were in arrears with their dues.

Dr. John H. Grant, Chairman of the Board of Censors, presented a report of work done by the Board since the last meeting, as follows:

*Memorandum report of the Board of Censors by Dr. John H. Grant, chairman, June 21, 1909.*

April 29.—The case of People vs. C. G. Edwards, "The Nature's Creation"—After submission to Grand Jury of evidence which appeared conclusive as to the unlawful practise of medicine by Edwards, the Grand Jury reported 'No Bill.' This was to be expected. The chairman of your board personally put in hard work in this case—addressed the Grand Jury, through the kindness of District Attorney Dudley—but it did not take long to find that he was facing a hostile foreman.

April 29.—The case of Wm. F. Bell mentioned in our last report as arraigned in the Police Court for illegally practising medicine was brought up April 29, and upon representations of his counsel that he was to file application with the Regents for an examination, the case was put over to June 10, to give Bell the desired opportunity. In the meantime, the chairman of your board communicated with the Regents Examination Division and was informed June 4, that they do not find William F. Bell has a license to practise medicine in this state, neither had he submitted the required evidence for admission to an examination. The case was again called in Police Court June 10, 1909, and upon your chairman refusing to consent to further delay, without a promise to the court on the part of the defendant to abstain from the further practice of medicine until lawfully authorised so to do, the defendant absolutely refused to so abstain and his attorney sprung certain objections—questioning the jurisdiction of the court, the form of the complaint and the law upon which it was based. Justice Nash said he would require time to look into these objections, and adjourned the case until June 18, on which date the case was further adjourned to July 1, defendant's counsel claiming Bell had received a card admitting him to the examination to be held here on or about June 21.

April 26.—On this date, a warrant was sworn out, in Police Court, against one, Mrs. C. Smith Paul of No. 382 Ellicott Square, Buffalo, for illegally practising osteopathy. In January last, Mrs. Paul was informed, by the chairman, that she would not be permitted to practise, as she was not registered in the Erie County Clerk's Office, she then was at 382 Ellicott Square and removed her sign "Dr. C. Smith Paul, Osteopathist" from the door. Later she moved to No. 387, and put on the door, a card in large letters, "Dr. C. Smith Paul, Osteopathist." She has a diploma from Kirksville, Mo., but has not sufficient counts (60) she says, to pass the Regents.

Arraigned in Police Court, April 27, case adjourned to April 29, on which date she plead guilty, and upon her promise to cease practising and arrange to go out of the state, your chairman recommended leniency and sentence was suspended.

May 6-7.—A young woman died in a hospital here of suspected abortion. An autopsy was held by the Medical Examiners at which the chairman of the board was present. Extensive gastroenteritis was found, also marked pericarditis and slight endocarditis, spleen pulp broken down, liver congested, womb showing evidence of recent evacuation of fetus, was found in fairly good condition, odor of iodoform, sections of the womb tissue were not made, although requested, and the medical examiner certified the cause of death as "gastroenteritis." As the family of the victim was strongly averse to any publicity, and no positive evidence being forthcoming, no further steps have been taken in the matter, although the board had a special meeting seeking evidence as to the perpetrator of the crime.

May 22.—Your chairman visited the office of Dr. Edward D. Porter, over 333 Main street, and insisted on examining the credentials of all there present which included Dr. Porter himself, two young men,—Arthur B. Cannon and Walter D. Register,—whose papers, including diplomas, Regents' licenses and County Clerk's registration appeared to be in proper form. Dr. Register laughingly said to your chairman, "You came near catching me, I have just registered. I heard you were after me." No females are treated by Dr. Porter, all being men patients, attracted by advertising. The prescriptions are put up upon the premises. Although this kind of practice is not to be commended, yet each and every one connected with it as named, are authorised by the state to practise medicine and surgery.

June 14.—An old offender—an alleged female abortionist—one, Mrs. Caroline Bailey, alias Jane Mullen, now located at Kenmore, on the confines of Buffalo, became such a stench to the good people of Kenmore, that several prominent persons complained to your chairman that a Mrs. Bailey, said to be a graduate nurse, was suspected of being engaged as an abortionist, on a prominent street of that village—automobiles with women stopping and going in there. A young married couple was engaged as detectives and after considerable visiting and talk with Mrs. Bailey and the expenditure of about $22.00, including a $10.00 fee, the attempt to perform an abortion on a young woman not pregnant and the introduction of a metal sound into the vagina and womb was made by Mrs. Bailey with instructions for the young woman to return in three days for a repetition of the job. Based upon the evidence thus obtained, application was made by sworn affidavit of the chairman to a Justice of the Peace at Kenmore, a Dr. Robert A. Toms, who absolutely refused, at first, to issue a warrant.

The chairman told Dr. Toms that it would be necessary to apply to the proper authorities for his removal from office, unless he performed his duty in the premises. He then called up the District Attorney and concluded to issue the warrant; also, upon my request, a search warrant, and with a Deputy Sheriff, the woman was arrested; the room in which the alleged abortion occurred was searched and the metal sound, the instrument used, was found and seized as evidence. On June 18, the case of Mrs. Bailey was heard before Justice Toms, she having plead not guilty on the day of arrest and bailed under a $1,500 bond. Justice Toms reserved decision but said he would render one in twenty-four hours.

In concluding this memorandum report, your chairman desires to say that he has been ably assisted in these cases by the advice and coöperation of Mr. Charles E. Doane, Counsel for the Society.

For the Board of Censors,

John H. Grant, Chairman.

Report was received and filed.

The recommendation made by the Council as to an outing to be held by the society was, on motion of Dr. Stockton, referred to the council with power to arrange for such outing.

The secretary presented the resignations of Dr. Edwin Janes and Dr. Albert R. Satterlee, both having removed from the county and from the state. The resignations were accepted. The secretary presented a communication from the secretary of the Medical Society of the State of New York, as follows:

At a meeting of the council held May 8, 1909, the following resolutions were passed:

*Resolved,* That on and after July 1, 1909, no member of the Medical Society of the State of New York shall receive the directory, the *New York State Journal of Medicine,* nor be entitled to malpractice defense until his county and state assessment has been paid.

*Resolved,* That in order to encourage increase in membership for the year 1909, all members who are elected between October 1, 1909, and December 31, 1909, and who shall pay during that period their state assessment may have the same credited to 1910, provided that they request it. All whose assessments are so credited shall be entitled to malpractice defense for 1909, but shall not be entitled to receive the directory, or the Journal for 1909. State assessments so credited shall be immediately forwarded by the County Treasurers to the State Treasurer.

It is very desirable from every standpoint that the membership be greatly increased during the year and we sincerely hope that your society will help us in this matter.

The communication was ordered, received and filed.

Dr. T. H. McKee, chairman of the committee on membership, presented the names of the following applicants each of whom was separately balloted for and duly elected: Elmer J. Wendel, 25 E. North street; Albert J. Harris, 633 E. Utica street; Jacob H. Dewees, 186 S. Park avenue; James E. King, 1248 Main street; C. Frank Bruso, 380 Main street; Herbert A. Smith, 106 Elmwood avenue; Edward G. Aldrich, Buffalo State Hospital; Cornelius J. Carr, 945 Genesee street; Edmund P. Reiman, 321 Pratt street; James Bailey Cross, 423 Potomac avenue; Edward H. F. Frisch, 888 Main street; William F. Jacobs, 89 Riley street; Bert J. Bixby, 82 Sycamore street; Carl Tompkins, 106 Elmwood avenue; Montressor Axford, 457 Breckenridge street; W. Harry Glenny, 185 Summer street; Frederick W. Koehler, 582 Broadway; Nahum Kavinoky, 576 Jefferson street; William W. Kitchen, 2612 Main street; Charles R. Cullinane, 180 Seneca street; William F. Twitty, 297 Woodlawn avenue; Nettie C. Heintz, 12 N. Pearl street; Isaac Sernoffsky, 889 Broadway, all of Buffalo.

Dr. Stockton stated that his attention had been called to a statement in the press to the effect that the Corporation Counsel had asked the Board of Health to present an ordinance relative the request of Treasurer Lytle, and on direction of the council, be instructed to confer with the Board of Health relative to the proposed ordinance regulating the distribution and sale of medicines throughout the city. Motion was carried.

Dr. Ullman, chairman of auditing committee stated that at the request of Treasurer Lytle, and on direction of the council, he had employed an auditor to go over the treasurer's accounts. He read the report which was adopted.

The scientific part of the program followed: Dr. William H. Billings read a paper on Relation of local skin lesion to flat foot, which was discussed by Drs. Meisenbach and McKee.

Dr. J. W. Putnam read a paper on Nervous sequelæ of infectious diseases. Discussed by Drs. Blaauw, Callanan, Bennett and Cohen.

Dr. Clayton Brown read a paper on Surgical importance of the vestibulary apparatus. Discussed by Drs. Blaauw, Benedict and Fairburn.

Dr. Myrtle L. Massey read a paper on Colon bacillaria. Discussed by Drs. Stockton, Cohen, Gardner, Wall and Hartwig.

Dr. James A. McLeod read a paper on Localised tuberculosis. Discussed by Drs. Bartow, Meisenbach and Blaauw.

A collation was served at the close of the meeting. There were present over 70 members.

